# Integrated analysis of miRNAs-mRNAs in skeletal muscle development revealed that novel-miR-766 affects myoblast differentiation and myofiber-type formation in sheep

**DOI:** 10.3389/fcell.2025.1615676

**Published:** 2025-07-31

**Authors:** Zhenzhen Gu, WeiWei Duan, Chenxi Liu, Wenrong Li, Bin Han, Mingjun Liu

**Affiliations:** ^1^College of Life Science and Technology, Xinjiang University, Urumqi, China; ^2^Key Laboratory of Genetics Breeding and Reproduction of Grass Feeding Livestock, Ministry of Agriculture and Rural Affairs, Xinjiang Academy of Animal Science, Urumqi, China; ^3^Key Laboratory of Animal Biotechnology of Xinjiang, Xinjiang Academy of Animal Science, Urumqi, China; ^4^Institute of Animal Biotechnology, Xinjiang Academy of Animal Science, Urumqi, China

**Keywords:** sheep, longissimus dorsi, miRNA, MYH3, myofiber

## Abstract

**Background:**

Various regulators coregulate muscle development in animals. MicroRNAs (miRNAs) are crucial regulators that participate in multiple aspects of myofiber formation. Method: To identify key miRNAs and target genes associated with muscle development, embryos or longissimus dorsi of Chinese Merino sheep were collected for whole-transcriptome sequencing across 11 gestation periods: 26 days (D26), D29, D32, D35, D40, D45, D55, D75, D85, D105, and D135. The functions of key miRNAs and target genes were determined by qRT-PCR, Western blot, dual-luciferase reporter gene assay, and Immunofluorescence staining.

**Results:**

A total of 284 differentially expressed miRNAs (DE-miRNAs) were screened by comparing the transcriptome data across all 11 periods. DE-miRNAs were divided into two developmental stages (stage A and stage B) based on heat map clustering analysis. Functional enrichment analysis revealed that DE-miRNAs in stage A were closely related to myofiber formation, whereas those in stage B were closely related to myofiber growth and maturation. Differential expression and functional enrichment analysis of target genes of DE-miRNAs obtained from stage A revealed the target relationships between 159 DE-miRNAs and 21 differentially expressed target genes associated with myofiber formation. In vitro assays revealed that myoblast differentiation and myotube formation were significantly inhibited by MYH3 knockdown via siMYH3 and that novel-miR-766 targets and decreases the expression of MYH3. In addition, the expression levels of marker genes related to myoblast differentiation and myofiber types were altered after the overexpression and inhibition of novel-miR-766 in sheep myoblasts.

**Discussion:**

This research not only elucidates the core temporal expression patterns of miRNAs but also suggests that novel-miR-766 influences myoblast differentiation and myofiber-type formation. This provides an important theoretical foundation for a deeper understanding of the molecular mechanisms by which miRNAs regulate myofiber development.

## Introduction

Myofiber development in sheep begins at the embryonic stage, and the number of myofibers throughout life is determined during the fetal stage. Postnatal muscle tissue development primarily relies on myofiber growth, including thickening and lengthening (Hindi et al., 2013). Therefore, elucidating the molecular mechanisms underlying sheep muscle fiber formation during fetal development holds significant theoretical and practical value for enhancing muscle production through genetic improvement strategies. Specific inactivation of Dicer during embryonic myogenesis results in perinatal lethality, reduced muscle mass, and abnormal muscle fibre morphology, suggesting that miRNAs are key regulators of embryonic muscle development (O'Rourke et al., 2007).

In recent years, significant progress has been made in regulation of skeletal muscle development by transcriptome studies in sheep, particularly in identifying key regulatory microRNAs and their target genes. Liu et al. (2019) analyzed the transcriptome data of longissimus dorsi muscle of Kazakh sheep in the fetal and yearling stages and identified 1,086 known miRNAs and 40 novel miRNAs. Among these, the target genes of the 345 differentially expressed miRNAs were significantly enriched in pathways related to skeletal muscle growth and development. Zhang et al. (2015) systematically investigated the miRNA expression profiles of skeletal muscle in Suffolk sheep across different developmental stages and revealed that miR-133 inhibits the proliferation of skeletal muscle satellite cells by targeting the TAGLN2, Rhoa, and CDC42 genes. Zhao et al. (2016) identified 2396 miRNAs and demonstrated that the expression of miR-192 tends to decrease during postnatal skeletal muscle development and satellite cell myogenic differentiation, regulating the myogenic differentiation process by targeting the RB1 gene.

However, the previous studies suffer from certain limitations. On the one hand, the temporal resolution is insufficient and is often confined to a few developmental stages. On the other hand, comprehensive experimental validation of the predicted miRNA‒mRNA interaction networks is lacking, particularly in functional studies of newly discovered miRNAs, which remain relatively weak. This study focuses on the Chinese Merino sheep breed, a dual-purpose sheep breed renowned for its high-quality wool and excellent meat quality. And innovatively conducts a systematic analysis of 11 consecutive gestational stages (D26–D135) in Chinese Merino sheep, constructing stage-specific regulatory networks for the myofiber formation phase (stage A) and the maturation phase (stage B) for the first time. By integrating dual-luciferase reporter assays, siRNA interference, and immunofluorescence techniques, this study provides the first experimental confirmation of the molecular mechanism by which novel miR-766 regulates muscle development by targeting MYH3. This research not only elucidates the core temporal expression patterns of miRNAs but also offers a crucial theoretical foundation for a deeper understanding of the molecular mechanisms by which miRNAs regulate muscle fiber type differentiation. The based on the regulatory network offer novel insights into the molecular mechanisms that govern.

## Materials and methods

### Ethics approval and consent to participate

All animal experiments in this study were approved by the Animal Ethics and Welfare Committee of the Institute of Animal Biotechnology of Xinjiang Academy of Animal Science (Permit No. SWS-B2017022011) and were conducted in accordance with the Regulations for the Administration of Affairs Concerning Experimental Animals (Ministry of Science and Technology, China, revised in March 2017). This study was conducted in accordance with all applicable institutional and governmental regulations.

### Animal selection and sample collection

The sheep used in this study were bred from a research flock of Chinese Merino sheep raised at the sheep breeding base of the Biotechnology Research Center of Xinjiang Academy of Animal Sciences. Adult Chinese Merino sheep of the same age, in good physical condition, and weighing 45–50 kg were selected for this study. Artificial insemination was performed after a uniform oestrus treatment period. The embryos were obtained through superovulation and subsequently transplanted into recipient ewes under the same physical conditions. Pregnant ewes were raised in accordance with the National Agricultural Industry Standards (NY/T816-2004). Ewes were humanely euthanized via a captive bolt pistol and exsanguinated at different gestation times. Fetuses were removed via cesarean section and euthanized with sodium pentobarbitone administered through cardiac puncture. The developmental stages are designated with “D” representing days, and the number indicates the number of days elapsed since fertilization. The embryos or longissimus dorsi tissues were collected from the fetus at the following gestational days: 26 days (D26) (five embryos), D29 (five embryos), D32 (four of unknown sex), D35 (five of unknown sex), D40 (four of unknown sex), D45 (two males and two females), D55 (one male and two females), D75 (one male and two females), D85 (three males), D105 (three males), and D135 (one male and three females).

### H&E staining

Longissimus dorsi tissues were fixed in phosphate-buffered 4% paraformaldehyde, embedded in paraffin, and sliced into 5 μm sections. The slices were immersed in xylene for 30 min and treated with 100% ethanol for 3 min. Then, treated with gradient ethanol of 95%, 90%, 80% and 70% for 1 min, respectively. Next, the slices were stained with hematoxylin for 10 min and eosin for 8 min to observe the muscle structure. Changes in muscle fiber number and diameter per bundle from D55-D135 (fiber number) or D75-D135 (diameter). The number and diameter of myofibers were manually counted under an optical microscope (OLYMPUS, Japan). Seven to ten sections were counted in each stage, and 3-5 muscle bundles were randomly counted in visual fields for each section. The error bars represent the standard deviations. The data are presented as the means ± SEM. Comparisons between two groups were performed using one-way ANOVA with GraphPad Prism 9.0 software.

### Total RNA isolation and sequencing

Total RNA was extracted from the samples taken from embryos or longissimus dorsi of Chinese Merino sheep fetuses (100 mg) via TRIzol reagent (Thermo, 1559026CN). The RNA integrity and concentration were assessed via an Agilent 2100 Bioanalyzer and NanoDrop ND-2000 spectrophotometer. Samples with RNA integrity numbers (RINs) above 7.0 were selected for sequencing. For mRNA sequencing, the RNase R enzyme is first used to cleave RNA into fragments of 250–300 bp. Random oligonucleotide primers, DNA polymerase I, and dNTPs are then used to synthesize cDNA. The cDNA was subjected to undergoes end repair, A-tailing, and sequencing adapter ligation, followed by PCR amplification to obtain the library. The Illumina HiSeq 4,000 sequencing platform from Novogene Bioinformatics Technology Co., Ltd. (Beijing, China) was used for double-terminal (pair-end) high-throughput sequencing. For small RNA sequencing, paired-end libraries were synthesized using the Small RNA Sample Prep Kit (Qiagen, Germany) according to the manufacturer’s instructions. The products were then purified and enriched via PCR to create the final cDNA library. The purified libraries were quantified with a Qubit® 2.0 fluorometer (Life Technologies, United States). Sequencing was performed, along with synthesis, using the Illumina SE50 platform (Illumina, United States).

### Analysis of sequencing data

The clean reads of the mRNA-seq data were mapped to the sheep reference genome (http://ftp.ensembl.org/pub/release-104/gtf/ovis_aries_rambouillet/) via HISAT2 software. Transcript assembly was performed using the StringTie software. Gene expression levels were estimated via the fragments per kilobase of transcript per million fragments (FPKM) method. DE-mRNAs were identified on the basis of a |log_2_(fold change)| ≥2 and adjusted *P* value (padj) <0.05. sRNAs with clean read lengths between 18-35 nt were screened. Bowtie software was used to map the screened sRNAs to the reference genome, with a mapping rate of greater than 70% considered acceptable. The reads mapped to the reference genome were compared with the sequences of known long noncoding RNAs, miRNAs, ribosomal RNAs, small nuclear RNAs, and transfer RNAs in the sheep mature miRNA database (https://mirbase.org) and the Rfam database (https://ftp.selab.janelia.org/pub/Rfam). The clean reads of miRNA-seq were mapped to the sheep mature miRNA database in miRBase (https://mirbase.org) and the Rfam database (https://ftp.selab.janelia.org/pub/Rfam) to align with known sequences of long noncoding RNAs, miRNAs, ribosomal RNAs, small nuclear RNAs, and transfer RNAs. Novel miRNAs were predicted via miREvo and miRDeep2. miRNA abundance was expressed as counts of transcripts per million mapped reads (TPM). DE-miRNA expression was based on a |log_2_(fold change)| ≥1 and adjusted *P* value (padj) < 0.05.

### miRNA target gene prediction, functional analyses, and network interactions

The tools of miRanda, TargetScan (http://www.TargetScan.org), and RNAhybrid (https://bibiserv.cebitec.uni-biele) were used to identify miRNA target genes. The GO (http://geneontology.org) and KEGG (http://www.genome.ad.jp/kegg/) databases were used to perform functional annotations. The display of relevant annotation results was performed via the OmicStudio tool at https://www.omicstudio.cn/tool. The network interactions between miRNAs and mRNAs were constructed via Cytoscape software (Smoot et al., 2011).

### siRNA design

The siRNA for MYH3 was designed and synthesized based on the sequence of MYH3 (XM_027974881.3) in the NCBI database by Shanghai Sangon Biological Engineering Co., Ltd. The siRNA sequence was 5′-GCAACTGGACACCAAGCTG-3'.

### Cell culture and transfer

Sheep myoblasts were used to verify the functions of MYH3 and the novel-miR-766. Primary myoblasts were derived from the longissimus dorsi of sheep on D45, following the protocol described by Gharaibeh et al. (2008). Myoblasts were cultured in growth medium (DMEM +20% fetal bovine serum +1% penicillin and streptomycin) on gelatin-coated plates in humidified incubators with 5% CO_2_ at 37°C. The cells were passaged when the confluence reached greater than 90%. They were then inoculated at a density of 1 × 10^6^ cells per well into a 6-well plate and cultured until they reached 80% confluence. Lipofectamine™ 3,000 was then used to transfect the myoblasts with siMYH3 (75 pmol/well), miRNA overexpression (25 pmol/well of novel-miR-766 mimics), knockdown (25 pmol/well of novel-miR-766 inhibitor), or negative controls (25 pmol/well of mimics-NC and inhibitor-NC) according to the manufacturer’s instructions. HEK-293T cells were used for the dual-luciferase reporter gene assay and cultured at a density of 1 × 10^6^/well in 6-well plates at 37°C in Dulbecco’s modified Eagle’s medium (DMEM) (Gibco, 11965092) supplemented with 10% fetal bovine serum (FBS) and 1% penicillin and streptomycin (Gibco, 15140122) in a humidified incubator with 5% CO_2_ (Thermo, United States). The cells were seeded into 12-well plates, and when they reached 80% confluence, Lipofectamine™ 3,000 (Invitrogen, L3000015) was used to cotransfect the cells with the constructed dual-luciferase reporter gene vectors (1 µg/well mirGLO-MYH3-WT, mirGLO-MYH3-MUT) along with novel-miR-766 (50 pmol/well mimic and mimic-NC).

### Immunofluorescence staining

The cells were fixed in 4% paraformaldehyde for 30 min and permeabilized in 0.2% Triton X-100 for 6 min at room temperature. The cells were blocked in 5% bovine serum albumin (BSA) for 30 min at 37°C and then incubated with a 1:200 primary antibody against Desmin (Sigma, D1033) at 4°C overnight, followed by incubation with a 1:200 Alexa Fluor 488-conjugated goat anti-mouse IgG (H + L) secondary antibody (Invitrogen, A11001) at room temperature for 1 h, with five washes with PBS washes after each antibody incubation. The cell nuclei were counterstained with DAPI (Solarbio, C0065). The immunofluorescence images were visualized with a fluorescence microscope (Nikon, Japan). Three photos with different visual fields were saved for each group of experiments, and the number of myotubes in the photos was manually counted and expressed as the mean ± SEM.

### Dual-luciferase reporter gene assay

Luciferase reporters used to verify the targeting relationships between miRNAs and mRNAs were generated based on the mirGLO vector (Promega, E133A). To construct mirGLO-MYH3, the complete 3′-UTR of sheep MYH3 mRNA (114 nt, GenBank accession no. XM_027974881.3), containing putative novel-miR-766 binding sites, was amplified, cloned and inserted into the mirGLO vector. The luciferase reporter was cotransfected with novel-miR-766 mimics and mimics-NC into 293T cells. Relative luciferase activity was measured via a Dual-Luciferase Reporter Assay System (Promega, E2940) and an Infinite M200 PRO microplate reader (Tecan, Swiss).

### qRT-PCR and Western blotting

Total RNA (tissues and cells) was extracted using TRIzol reagent and then reverse-transcribed using the miRNA 1st strand cDNA synthesis kit (Accurate, AG11717) and the Fast King RT Kit (TIANGEN, China), following the manufacturer’s instructions. qRT-PCR was used to quantify the expression levels of novel-miR-766, MYH3, MYH7, MYH2, and MYH4 in different treatment groups using Fast SYBR™ Green Master Mix (Roche, 4913914001) according to the manufacturer’s instructions. The primer amplification efficiency was within the range of 90%–100%, meeting the requirements for determining mRNA relative abundance using the 2−ΔΔCt method. ANOVA was used for the statistical comparison of more than two groups, with *P* < 0.05 considered statistically significant. For Western blot analysis, the myoblasts were lysed with RIPA lysis buffer II (Thermo, 89,900). The protein concentrations in the different groups were measured with a Pierce™ BCA protein assay kit (Thermo, 23,227). Proteins (20 µg/lane) were separated via SDS-PAGE, transferred to polyvinylidene difluoride membranes, and probed with mouse anti-MYH3 (dilution 1:2000, Abcam, ab264038), rabbit anti-actin (dilution 1:2000, Abmart, T40001), mouse anti-MYOG (dilution 1:1000, Abcam, ab1835), and mouse anti-MYOD (dilution 1:1000, Abcam, ab16148) antibodies. After incubation with either goat anti-rabbit IgG (H + L) (dilution 1:1000, Beyotime, A0208) or HRP-conjugated goat anti-mouse IgG (H + L) (dilution 1:2000, Beyotime, A0216) antibodies, signals were detected via an enhanced chemiluminescence (ECL) substrate kit (Biosharp, BL520A) and visualized with AI600 imaging instruments (GE, United States). The band intensities of the target proteins were quantified via ImageJ software, with β-actin serving as the internal reference protein.

## Results

### Histologic features of myofiber development in sheep fetuses

**FIGURE 1:**
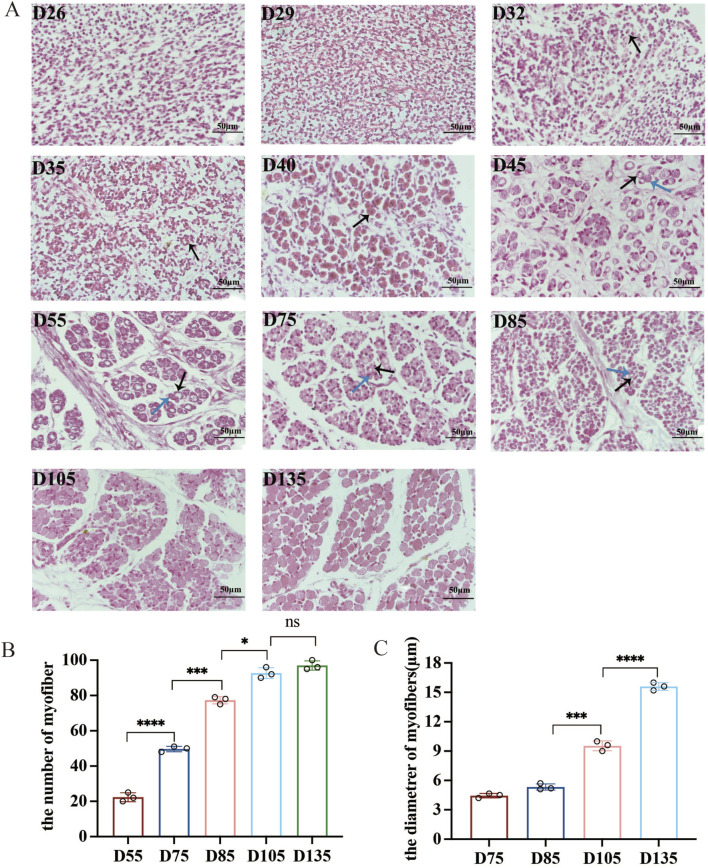
Dynamics of myofiber development in sheep fetuses (400×). **(A)** Primary and secondary myofibers are indicated with black and blue arrows, respectively. **(B,C)** Changes in muscle fiber number and diameter per bundle from D55-D135 (fiber number) or D75-D135 (diameter). NS (not significant), *P* > 0.05, *P < 0.05, ***P < 0.001, and ****P < 0.0001.

In this study, 11 Chinese Merino sheep with the longissimus dorsi muscles at different embryonic developmental stages were subjected to transverse paraffin sectioning and HE staining to observe the phenotypic changes in the process of muscle fibre formation (Figure 1A). Myofiber formation was not observed at D26 and D29 of the embryonic stage. Primary fibres started to form from D32 onward, with the number of primary fibres reaching a peak at D40. A large number of secondary fibres formed between D45 and D85 (Figure 1A). The number of myofibers remained essentially constant after D105, but the length and diameter of the myofibers continued to increase, and the area and diameter of the myofibers expanded significantly from D105 to D135, a critical period for the development of myofiber morphology and function (Figures 1B,C).

### Overview of the small RNA sequencing data and differential expression analysis

**FIGURE 2:**
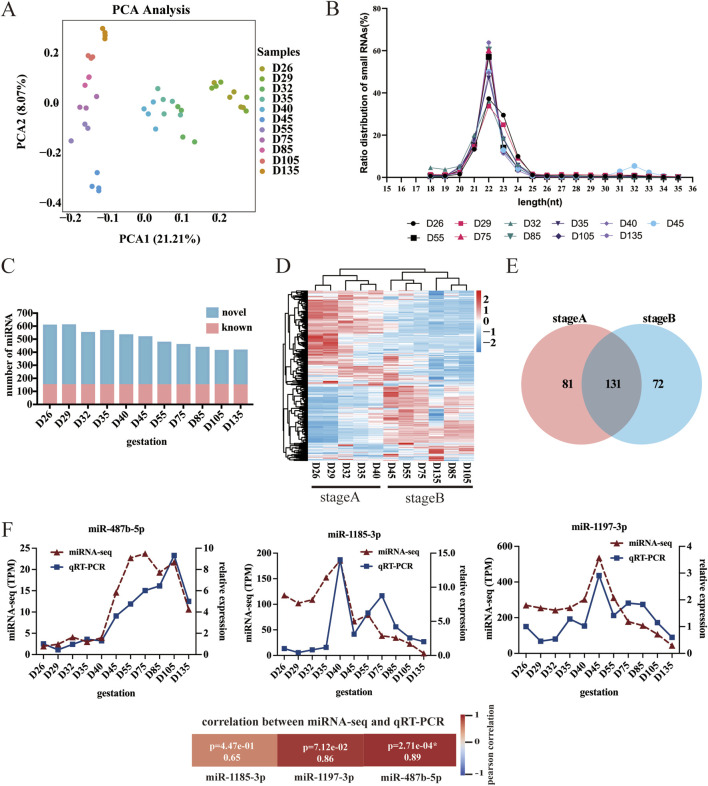
Characteristics of the miRNA data. **(A)** Principal component analysis (PCA) of miRNAs. **(B)** MiRNA length analysis. **(C)** miRNA number analysis. **(D)** Heatmap of DE-miRNAs. **(E)** Venn diagram showing DE-miRNAs. **(F)** Analysis of miRNA expression levels via qRT-PCR.

In this study, the raw data of 43 sequenced libraries were analyzed for quality control (QC), and more than 97.12% of the raw reads met the quality control (QC) standard. The Q30 value ranged from 92.13% to 97.67%, the GC content was approximately 50%, and the balanced distribution of bases was in accordance with the theoretical distribution ratio. To ensure the accuracy of the subsequent analysis, principal component analysis (PCA) was performed on the sequencing results of 43 samples (Figure 2A). PCA revealed that the samples from the same period essentially clustered together, which indicated that the samples were reproducible and could be used for subsequent analysis. The miRNA fragments with a conserved length of 22 nt accounted for the largest proportion (Figure 2B). A total of 152 known miRNAs and 633 novel miRNAs were identified. During fetal development, the total abundance of miRNAs gradually decreases. Notably, the levels of known miRNAs remained stable, whereas those of newly identified miRNAs declined progressively (Figure 2C). To validate the sequencing data, three randomly selected miRNAs were analyzed using qRT-PCR. The qRT-PCR results correlated well with the miRNA-seq data (Figure 2F), confirming the accuracy and reliability of the sequencing results.

To investigate the regulatory role of miRNAs in sheep myofiber formation and development, we conducted pairwise comparisons of miRNAs across different developmental stages and identified 284 differentially expressed miRNAs (DE-miRNAs). Specifically, the greatest number of DE-miRNAs (216) was detected between D26 and D135, and the lowest number (zero) was detected between D26 and D29 (Supplementary Figure S1). Cluster heatmap analysis of all the differentially expressed miRNAs revealed that the DE-miRNAs could be categorized into stage A (the beginning of myogenesis and the formation of primary fibers, i.e., D26, D29, D32, D35, and D40) and stage B (the formation of secondary myofibers and the maturation of myofibers, i.e., D45, D55, D75, D85, D105, and D135) (Figure 2D). Next, we generated Venn diagrams for stage A and B. There were 212 stage A DE-miRNAs and 203 stage B DE-miRNAs (Figure 2E).

### Functional enrichment of DE-miRNAs

**FIGURE 3:**
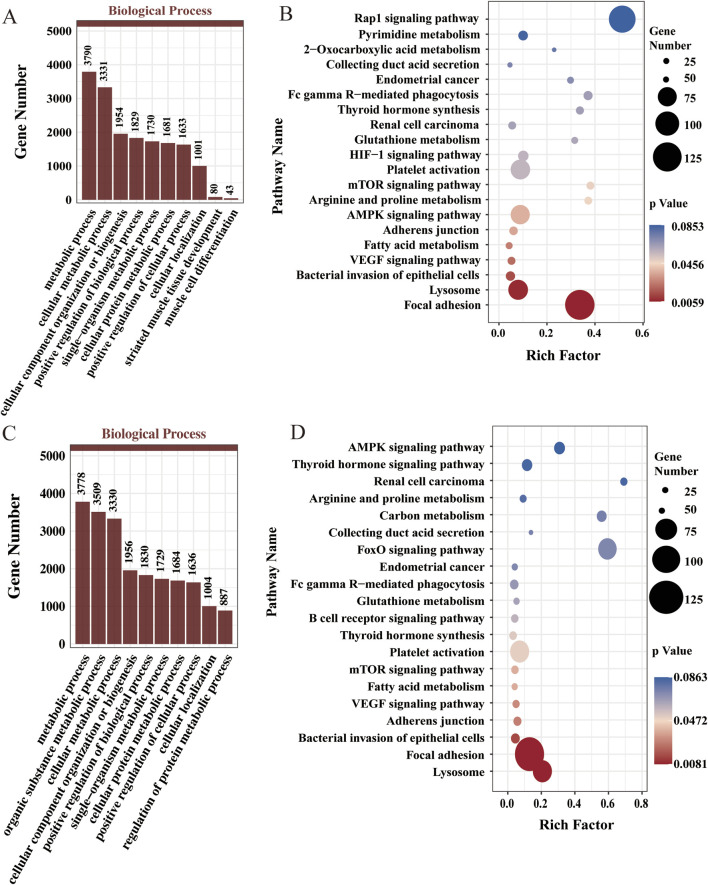
Functional enrichment analysis for DE-miRNAs. **(A)** GO enrichment analysis for DE-miRNAs in stage A (top 10 terms in the biological process). **(B)** KEGG enrichment analysis results for the DE-miRNAs in stage A (top 20). **(C)** GO enrichment analysis of DE-miRNAs in stage B (top 10 terms in the biological process category). **(D)** KEGG enrichment analysis results for the DE-miRNAs in stage B (top 20).

We performed GO and KEGG functional enrichment analyses on the target genes of differentially expressed miRNAs across various stages to assess their biological functions and identify those associated with muscle development in Chinese Merino sheep. The target genes of the DE-miRNAs in stage A were significantly enriched in terms related to myofiber formation, such as muscle cell differentiation, striated muscle tissue development, cellular component organization or biogenesis, and protein complex binding. These genes were enriched in pathways, including fatty acid metabolism, 2-oxocarboxylic acid metabolism, the AMPK signaling pathway, and the mTOR signaling pathway (Figures 3A,B). In stage B, the target genes of the DE-miRNAs were significantly enriched in terms related to myofiber growth and maturation, such as metabolic processes, cells, protein complex binding, and catalytic activity, as were pathways related to carbon metabolism, the FoxO signaling pathway, fatty acid metabolism, the AMPK signaling pathway, and the mTOR signaling pathway (Figures 3C,D).

### Construction of the mRNA-miRNA network

To better understand the regulatory roles of miRNAs in sheep myofiber formation, we performed differential expression analysis of the target genes of the DE-miRNAs obtained. Six hundred seventy-nine of the target genes in stage A were DE-mRNAs, and 684 of the target genes in stage B were DE-mRNAs. The above functional enrichment analysis indicated that the genes in stage A were significantly enriched in biological processes and pathways related to muscle formation. Therefore, DE-mRNAs and DE-miRNAs in stage A were selected to construct the interaction network.

**FIGURE 4:**
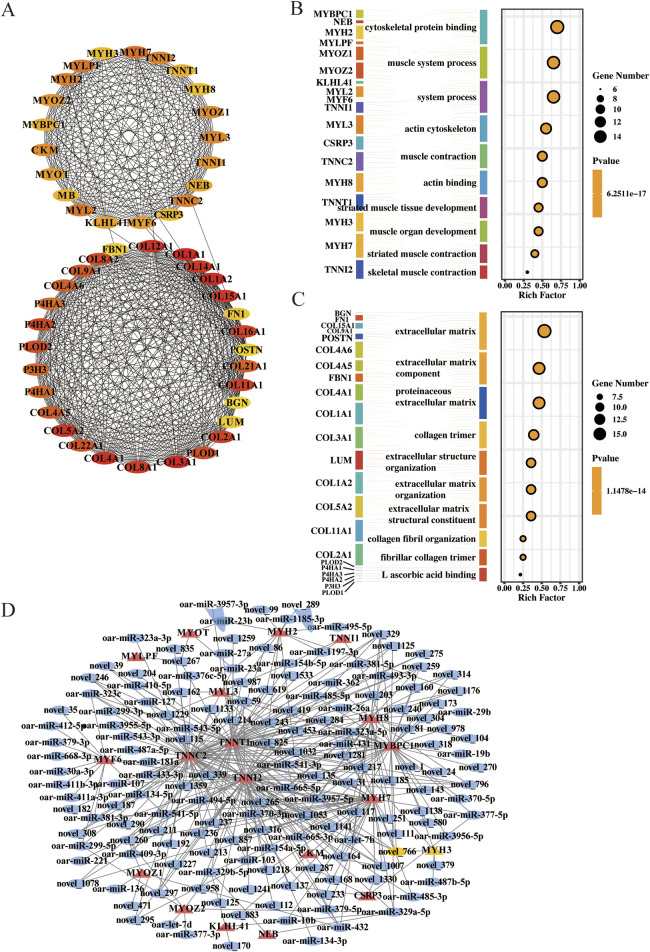
Network of DE-mRNAs and DE-miRNAs in stage **(A)**. **(A)** Top 50 core target genes in stage **(A)**. **(B)** Top 10 terms of GO enrichment analysis for 21 genes. **(C)** Top 10 terms of GO enrichment analysis for 29 genes. **(D)** Key miRNAs and mRNAs involved in myofiber formation. Circle: miRNA; triangle: mRNA.

Among the 679 mRNAs, the top 50 core genes were identified (Figure 4A). These 50 genes were divided into two groups based on their interactions: one group of 21 mRNAs, including MYH3, and the other group of 29 mRNAs, including COL12A1. GO functional enrichment analysis of these two groups of genes revealed that 21 mRNAs were significantly enriched in terms related to muscle development, including skeletal muscle contraction and processes within the muscular system (Figure 4B). Twenty-nine mRNAs related to the extracellular matrix and collagen trimerization were enriched (Figure 4C). Finally, 21 genes were selected for subsequent studies to construct the interaction network of stage A DE-mRNAs and DE-miRNAs (Figure 4D).

### MYH3 promotes myoblast differentiation and enhances myotube formation

**FIGURE 5:**
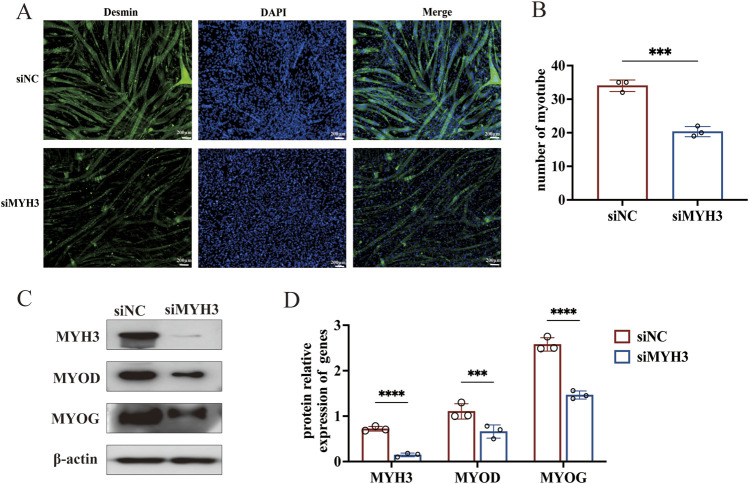
MYH3 promotes the differentiation of myoblasts and myotube formation in sheep. **(A)** Myotube formation was observed by immunofluorescence staining for desmin (100×). **(B)** Myotube quantification. **(C)** Changes in the expression of MYOD and MYOG after interference with MYH3. **(D)** Analysis of the relative protein expression of MYH3, MYOD and MYOG. ****P < 0.001, ***P < 0.005.

To demonstrate the applicability of the approach used in this study, we focused on one of the DE-miRNAs, novel-miR-766, and its target gene, MYH3. Studies in mice have demonstrated that MYH3 is a key marker gene for muscle differentiation. It regulates muscle development and energy metabolism, playing a vital role in the overall muscle development process in animals (Isobe et al., 2018; Sala et al., 2019; Agarwal et al., 2020). To determine the role of MYH3 in sheep muscle development, we utilized siRNA to investigate its impact on myoblast differentiation. First, sheep myoblasts were transfected with a synthetic siRNA targeting MYH3. After 48 h, the growth medium was replaced with a differentiation medium to induce cell differentiation. After 4 days of differentiation, the cells were fixed for the immunofluorescence assay. Differentiated myotubes were labelled and stained with an antibody against desmin, and images were captured using an inverted fluorescence microscope (Figure 5A).

The results of the immunofluorescence assay indicated that, compared with control cells, cells transfected with siMYH3 formed fewer myotubes. The number of myotubes was quantified by counting those in at least nine randomly selected visual fields from each group, confirming that the number of myotubes formed by the siMYH3-transfected cells was significantly lower than that formed by the control cells (Figure 5B). Western blotting was used to detect changes in the expression of MYOD and MYOG (marker genes of myoblast differentiation) after MYH3 knockdown. The expression levels of MYOD and MYOG were significantly lower than those in the control group, confirming the enhancing effect of MYH3 on myoblast differentiation in sheep (Figures 5C,D).

### Validation of the targeting relationship between MYH3 and novel-miR-766

**FIGURE 6:**
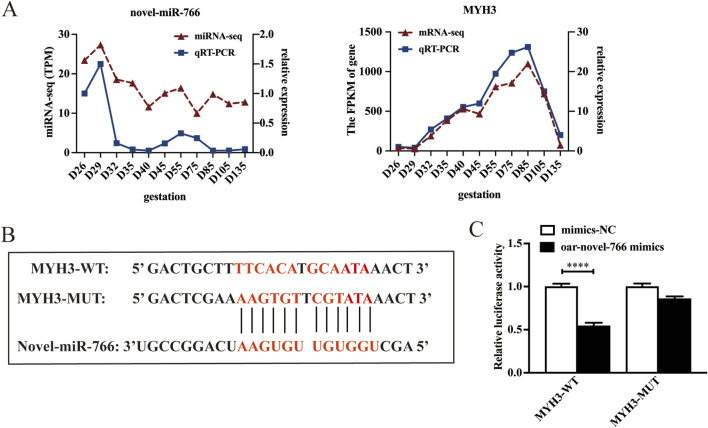
Novel-miR-766 targets MYH3 expression. **(A)** qRT-PCR and RNA-seq analysis of the mRNA expression of novel-miR-766 and MYH3. **(B)** Predicted binding site between novel-miR-766 and MYH3. **(C)** A luciferase reporter gene assay revealed that the overexpression of novel-miR-766 significantly quenched the fluorescence of wild-type MYH3. ****P < 0.001.

In this study, seven miRNAs were predicted to target MYH3 (Figure 4D). Among these, novel-miR-766 showed downregulated expression in the longissimus dorsi of sheep fetuses across all developmental stages, exhibiting an inverse trend compared with its target gene MYH3. These findings suggest a potential antagonistic relationship between novel-miR-766 and MYH3. To validate this finding, we analyzed the expression patterns of novel-miR-766 and MYH3 in sheep skeletal muscle at different developmental stages via qRT-PCR (Figure 6A), which revealed that the expression trends of novel-miRNA-766 and MYH3 were opposite. To determine whether novel-miR-766 targets and regulates MYH3, a luciferase reporter vector (pmirGLO-MYH3) was constructed and transfected into 293T cells. MYH3 luciferase reporters with wild-type and mutated MYH3 3′-untranslated regions (UTRs) were constructed (Figure 6B). These reporters were cotransfected with novel-miR-766 mimics or negative control (mimic-NC) into 293T cells. Luciferase reporter activity was measured via dual-luciferase assays. Luciferase activity decreased in cells cotransfected with novel-miR-766 mimics compared with those transfected with the mutant or empty controls. The overexpression of novel miR-766 mimics reduced the luciferase activity of pmirGLO-MYH3 (Figure 6C). Thus, MYH3 is indeed regulated by novel-miR-766.

### Novel-miR-766 regulates myoblast differentiation and myofiber specification by targeting MYH3

**FIGURE 7:**
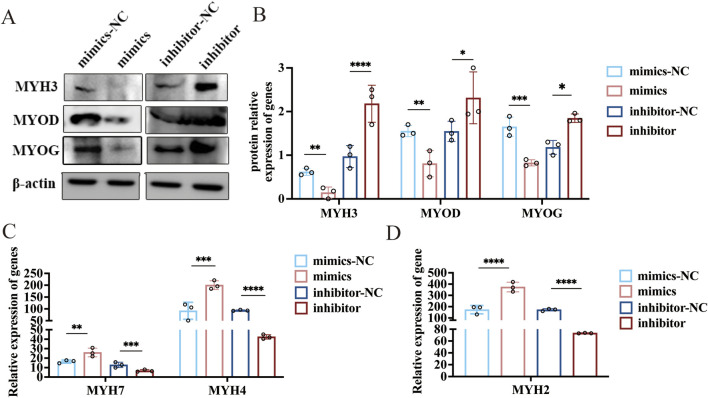
The novel-miR-766 regulates myoblast differentiation and myofiber specification. **(A)** Western blot analysis of MYH3, MYOD, and MYOG expression levels after the overexpression and inhibition of novel-miR-766. **(B)** Analysis of the relative protein expression of MYH3, MYOD, and MYOG. **(C–D)** qRT-PCR analysis of MYH7, MYH4, and MYH2 expression levels after the overexpression and inhibition of novel-miR-766. ****P < 0.001, ***P < 0.005, **P < 0.01, *P < 0.05.

Sheep myoblasts were transfected with novel-miR-766 mimics and inhibitors to examine the effect of novel-miR-766 on myoblast differentiation. After 48 h of transfection, the cells were collected, and the total protein was extracted. The expression levels of MYH3, MYOD, and MYOG were assessed via Western blotting to evaluate the effects of overexpressing and inhibiting novel-miR-766 (Figure 7). The expression levels of MYH3, MYOG, and MYOD in cells transfected with the inhibitor were higher than those in the control group (*P* < 0.05), whereas the opposite trend was observed in cells transfected with mimics. Thus, novel-miR-766 inhibited myoblast differentiation.

MYH3 regulates the size, number, and type conversion of myofibers (Agarwal et al., 2020), and high MYH3 expression is associated with the content of oxidized type-I myofibers, the diameter of myofibers, and the content of slow myofibers in skeletal muscles (Cho et al., 2019). Therefore, we explored whether novel-miR-766 could affect the type conversion of myofibers in sheep. After transfection of sheep myoblasts with novel-miR-766 mimics and inhibitors, along with their respective controls, the cells were induced to differentiate for 96 h and then collected to extract RNA. qRT-PCR analysis revealed that the slow myofiber marker gene MYH7 and the fast-twitch myofiber marker genes. MYH2 and MYH4 were significantly upregulated in mimic-transfected cells compared with control cells, with MYH2 showing the most pronounced increase. Both fast- and slow-twitch myofiber marker genes were significantly downregulated in inhibitor-transfected cells (Figures 7C,D). These findings demonstrate that the novel-miR-766 regulates the expression of fast- and slow-twitch myofiber marker genes in ovine myoblasts, thereby modulating myofiber-type specification and conversion.

## Discussion

Given the vital role of miRNAs in muscle development, numerous recent studies have investigated the types of miRNAs expressed in skeletal muscles, their target genes, and their functions in myogenesis across various species over the past decade. RNA-sequencing and ChIP technologies have been employed to analyze miRNA profiles in the skeletal muscles of pigs (Huang et al., 2008; Xie et al., 2010; Siengdee et al., 2013), horses (Kim et al., 2014), goats (Wang et al., 2014), and cows (Muroya et al., 2013). Consequently, many novel miRNAs have been discovered, laying a solid foundation for future research. This study revealed that DE-miRNAs can be divided into two developmental stages (stage A and stage B). This finding is consistent with previous studies in which a similar pattern of stage-specific regulation has been observed in rat muscle development (Ross et al., 1987). We further refined the molecular features of these two stages through functional enrichment analysis and revealed the key role of novel-miR-766 in stage A.

Research on miRNA functions typically involves overexpression or interference (inhibition and knockdown). For example, Voellenkle et al. (Bogerd et al., 2010) confirmed the effects of novel miRNAs on cell growth through overexpression studies. This study established a cellular model for the overexpression and inhibition of novel-miR-766 through transfection with mimics and inhibitors, respectively. Specifically, endogenous MYH3 expression was reduced in myoblasts following transfection with the novel-miR-766 mimic. Concurrently, myoblast differentiation marker genes were significantly downregulated, and myotube formation was inhibited, suggesting that novel-miR-766 suppresses myoblast differentiation.

In mice, the overexpression of MYH3 can lead to an upregulation of MYH7 (Cho et al., 2019). MYH3 may have different functions in different muscles, with genes physically closer to MYH3 tending to be upregulated in a broader range of muscles. In a mouse model of MYH3 deletion, the transcription levels of MYH1 were elevated in the quadriceps and diaphragmatic muscles, whereas those of MYH8 and MYH2 were significantly elevated in the quadriceps and gastrocnemius muscles. In contrast, the transcription levels of MYH7 are decreased (Agarwal et al., 2020). In this study, we found that the expression of MYH3 decreased after the overexpression of novel-miR-766 in sheep myoblasts. Moreover, the slow myofiber marker gene MYH7 and the fast myofiber marker genes MYH2 and MYH4 were significantly upregulated. These findings suggest that novel-miR-766 may play a role in determining myofiber type. This finding carries important implications, as existing research indicates that muscle cell differentiation indirectly shapes the flavor, tenderness, and juiciness of meat by determining muscle fiber type composition and metabolic characteristics (Picard and Gagaoua, 2020; Sirin and Sen, 2018). Our study is the first to reveal the regulatory role of the novel-miR-766-MYH3 axis in myofiber type specification, providing not only new insights into miRNA function during muscle development but also establishing a theoretical foundation for future genetic approaches to modulate fiber-type conversion for meat quality improvement.

Although our functional analysis focused on the novel-miR-766-MYH3 axis, the coexpression network identified 20 additional mRNAs that may be involved in sheep muscle fibre formation. Notably, a literature review revealed that these genes play well-defined roles in muscle development in different species. For example, MYL2 encodes myosin light chain 2, which is involved in sarcomere assembly and the regulation of muscle contraction (Yahya et al., 2022; Hoh, 2023; Kumar et al., 2023). CKM (creatine kinase M type) plays a central role in energy metabolism, providing ATP for muscle contraction (Borzemska et al., 2024). KLHL41 participates in muscle fiber formation by regulating myofibril assembly (Pak, 2019). MYF6, as a myogenic regulator, regulates muscle cell terminal differentiation and muscle fiber maturation (Sabourin and Rudnicki, 2010). The NEB gene encodes an actin-associated protein, an important component of thin filaments (Zhang et al., 2024; Zídková et al., 2025). MYOT acts as an intermediate filament protein to maintain the structural integrity of muscle fibers (Guglielmi et al., 2023). MYLPF encodes myosin light chain phosphatase, which regulates muscle contraction (Brunello and Fusi, 2024). CSRP3, a Z-disk component protein, plays a role in maintaining sarcomere structure (Li et al., 2022). MYBPC1 regulates the assembly and function of thick filaments (Bhandari et al., 2024). Different myosin heavy chain genes (MYH2, MYH3, MYH7, and MYH8) encode specific subtypes of fast and slow muscle fibres, which determine the characteristics of muscle fibre types (Picard and Gagaoua, 2020; Blemker et al., 2024). MYL3 participates in the formation of the formation of myosin complexes (Schiaffino and Reggiani, 2011). MYOZ1 and MYOZ2, as Z-disk-associated proteins, participate in mechanical signal transduction (Fatchiyah et al., 2022; Zhou et al., 2022). Members of the troponin complex (TNNC2, TNNI1, TNNI2, and TNNT1) regulate calcium ion sensitivity to finely modulate muscle contraction (Ogasawara and Nishino, 2021; van de Locht et al., 2021). These genes act synergistically to collectively regulate the formation, differentiation, and functional specialization of muscle fibers. Although further experimental validation is necessary, these genes represent high-priority candidate genes for future research. The predicted interactions of these miRNAs with stage-specific miRNAs suggest that a broader regulatory network governing sheep muscle development requires systematic exploration.

Current studies on novel-miR-766 still have several limitations. First, cell-based experiments using novel-miR-766 mimics/inhibitors may not fully replicate the *in vivo* context, potentially leading to discrepancies from physiological conditions. Second, this study has not yet employed cotransfection experiments (such as simultaneously introducing novel-miR-766 mimics and MYH3 overexpression vectors) to validate their dynamic regulatory relationship in myocytes, which limits our in-depth understanding of their interaction mechanisms. Third, this study focused only on the direct interaction between novel-miR-766 and its target gene MYH3, neglecting other regulatory networks, such as the ceRNA network or epigenetic regulation, which may limit a comprehensive understanding of the biological roles of novel-miR-766. Additionally, reliance on a single type of experimental data (e.g., dual-luciferase assays or qRT-PCR) without integrating multi-omics may hinder the construction of novel miR-766 regulatory networks and the acquisition of functional insights. In future studies, we will conduct more accurate and comprehensive investigations into the biological function of novel-miR-766 *in vivo* and *in vitro*, taking into account the above limitations.

In addition, recent studies (Wagner et al., 2024; Ehsan et al., 2020; Corine et al., 2012) demonstrate that specific miRNA isoforms (isomiRs) exhibit distinct expression patterns and target preferences compared to their canonical miRNA counterparts, potentially contributing to the fine-tuning of myogenic differentiation and myofiber-type specification. While our current study focused on canonical miRNA-mRNA interactions, we explicitly recognize that future work incorporating isomiR analysis could provide additional mechanistic insights into the complex regulatory networks governing skeletal muscle development.

## Conclusion

In summary, this study identified key miRNAs associated with myofiber formation and constructed a miRNA-mRNA regulatory network that is potentially involved in this process. Furthermore, we demonstrated that novel-miR-766 negatively regulates MYH3 expression, thereby influencing sheep myoblast differentiation and the expression of myofiber-type marker genes. These findings provide novel insights into the molecular mechanisms underlying myofiber formation in sheep.

## Data Availability

The datasets presented in this study can be found in online repositories. The names of the repository/repositories and accession number(s) can be found below: https://www.ncbi.nlm.nih.gov/, Nos PRJNA1083059 and PRJNA1267602.

## Supplementary material

SUPPLEMENTARY FIGURE S1 DE-miRNAs from the two groups obtained via pairwise comparison.
