# Supplementary figures and images for: Integrated analysis of miRNAs-mRNAs in skeletal muscle development revealed that novel-miR-766 affects myoblast differentiation and myofiber-type formation in sheep
Source: Front Cell Dev Biol. 2025 Jul 31;13:1615676. doi: 10.3389/fcell.2025.1615676 (PMC12350436; doi:10.3389/fcell.2025.1615676)

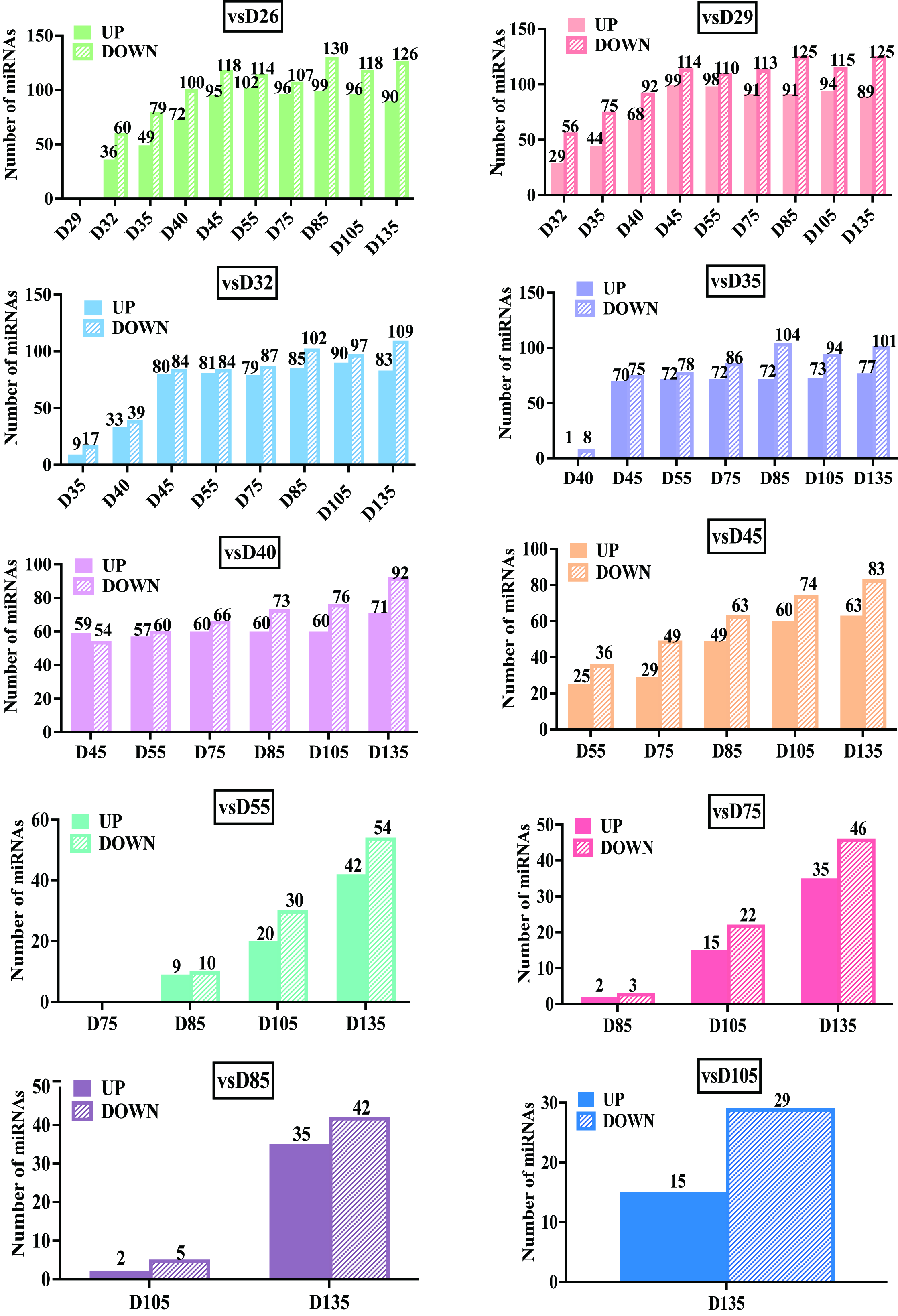

Supplement: Supplementary file 3 [file Image1.tif]
